# Surfactantless Emulsions Containing Eugenol for Imidacloprid Solubilization: Physicochemical Characterization and Toxicity against Insecticide-Resistant *Cimex lectularius*

**DOI:** 10.3390/molecules25102290

**Published:** 2020-05-13

**Authors:** Mariano Cáceres, Eduardo Guzmán, Agustín Alvarez-Costa, Francisco Ortega, Ramón G. Rubio, Carlos Coviella, Pablo L. Santo Orihuela, Claudia V. Vassena, Alejandro Lucia

**Affiliations:** 1 Centro de Investigaciones de Plagas e Insecticidas (UNIDEF–CITEDEF, CONICET), San Juan Bautista de La Salle 4397, Villa Martelli 1603, Buenos Aires, Argentina; psorihuela@gmail.com (P.L.S.O.); cvassena@gmail.com (C.V.V.); 2 Departamento de Química Física, Facultad de Ciencias Químicas, Universidad Complutense de Madrid, Ciudad Universitaria s/n, 28040 Madrid, Spain; fortega@quim.ucm.es (F.O.); rgrubio@quim.ucm.es (R.G.R.); 3 Instituto Pluridisciplinar, Universidad Complutense de Madrid, Paseo Juan XXIII, nº1, 28040 Madrid, Spain; 4 Departamento de Biodiversidad y Biología Experimental, Facultad de Ciencias Exactas y Naturales, Universidad de Buenos Aires, Ciudad Universitaria, Ciudad Autónoma de Buenos Aires 1428, Argentina; agustinalvarezcosta@gmail.com; 5 Instituto de Ecología y Desarrollo Sustentable (INEDES, CONICET-UNLu), Ruta 5 y Avenida Constitución, Luján 6700, Buenos Aires, Argentina; carlosecoviella@yahoo.com; 6 Departamento de Ciencias Básicas, Universidad Nacional de Lujan, Ruta 5 y Avenida Constitución, Luján 6700, Buenos Aires, Argentina; 7 Cátedra de Química Analítica Instrumental, -Facultad de Farmacia y Bioquímica, Universidad de Buenos Aires, Junín 954, Ciudad Autónoma de Buenos Aires 1113, Argentina; 8 Instituto de Ingeniería e Investigaciones Ambientales, Universidad Nacional de San Martín, Campus Miguelete, 25 de Mayo y Francia, San Martín 1650, Buenos Aires, Argentina; 9 Centro de Investigación en Sanidad Vegetal (CISaV), Facultad de Ciencias Agrarias y Forestales, Universidad Nacional de La Plata, Calles 60 y 119, La Plata 1900, Buenos Aires, Argentina

**Keywords:** eugenol, surfactantless emulsions, ternary mixtures, imidacloprid, bed bugs

## Abstract

Synthetic insecticides have been used for a long time as one of the most effective tools for insect pest control. However, the re-emergence of insect pests and their fast development of resistance, as has occurred for pyrethroid-resistant bed bugs *Cimex lectularius* L., make it necessary to develop new and safe strategies for effective pest control. This has fostered the research on new eco-sustainable formulations based on essential oils, which allows reducing the impact associated with the intensive use of synthetic insecticides on the environment and their effects on human health. This research explores the stability of water/eugenol/ethanol surfactantless emulsions loaded with imidacloprid (0.003 wt%), and their toxicity against a resistant bed bug strain. The results have shown that these emulsions enable the solubilization of a poorly water-soluble drug, such as the imidacloprid, without any significant modification of their stability. Furthermore, the application of the obtained formulations against the pyrethroid-resistant bed bug results in mortality in the 50–85% range upon topical and spray applications, with the increase of the eugenol content enhancing the effectiveness of the formulations. It may be expected that the ternary water/eugenol/ethanol mixtures could be further developed in the preparation of ready to use formulations, enabling the dispersion of insecticides for pest control.

## 1. Introduction

Essential oils (EOs) are lipophilic secondary metabolites obtained from aromatic plants, with terpenoids being their main components, which present insecticidal active [1,2,3]. This plays an important role in the development of new environmentally friendly [4,5] and safer for human and non-target species [6] formulations for insect pest control. However, the number of practical applications of botanical insecticides remains rather limited yet (below 5% of the global market) [7]. This is, in part, a result of their poor solubility in water and their chemical instability, which make their storage and handling difficult. Furthermore, the introduction of new botanical insecticides for insect pest management requires a careful examination of the most appropriate control strategies to prevent the rapid onset of insecticide resistance [8,9], which is one of the main problems associated with any product used in pest control programs [10]. This becomes clearer with a closer look at the use of DDT (dichlorodiphenyltrichlorethane) and pyrethroids, which may be regarded as the most successful insecticides for insect pest control. The intensive use of DDT during the 1950s and 1960s, and its progressive substitution by pyrethroids in the 1970s, resulted in the emergence of high levels of resistance between different insect species [11]. Currently, neurotoxic insecticides are widespread for insect pest control, with pyrethroids and neonicotinoids being the most commonly used (43% of the global insecticide sales) [12,13].

The recent re-emergence of the human parasite *Cimex lectularis *L. and the fast development of its resistance to pyrethroids have required the design of new strategies for controlling the bed bug populations, with the combination of neonicotinoids and pyrethroids being the most recommended strategy for facing this insect pest [14,15,16,17,18,19]. However, the extensive use of such formulations has resulted in a significant reduction of their effectiveness in dry residual and contact surface treatments [20,21,22,23,24]. A recent study has assessed the susceptibility of bed bug colonies collected in Argentina to imidacloprid (neonicotinoids) and deltamethrin (pyrethroids), with resistance ratios to such insecticides being higher than those corresponding to the susceptible strain (up to 54- and 40,000-fold for pyrethroids and neonicotinoids, respectively) [20,25,26,27].

Therefore, since new formulations would help to circumvent the resistance, the use of insecticides containing EOs or their compounds is one of the most promising alternatives for facing the problems associated with such resistance [28,29,30,31].

The use of EOs in pesticide formulations may be used either as active ingredient of the formulation or as adjuvants. The latter case is of particular interest because several studies have reported synergistic effects against insect pests of mixtures containing EOs or its compounds combined with synthetic insecticides [32,33,34,35,36,37]. The emergence of such synergistic effects might be associated with two characteristics of terpenoids: (i) enhancers of the penetration of both lipophilic and hydrophilic compounds [38], and (ii) inhibitors of metabolic enzymes such as cytochrome P450 monooxygenases and carboxylesterases [32].

This research explores the potential synergistic effects against bed bugs resistant to formulations containing an essential oil (eugenol) and a synthetic insecticide belonging to the neonicotinoids family (imidacloprid). This will exploit the ability of surfactantless emulsions for drug solubilization due to the hydrotropic to solubilize that enables the dispersion of the imidacloprid within a liquid dispersion which may enhance the distribution of this poor water-soluble insecticide in the environment [39,40,41,42]. It is worth noting that the surfactant–free character of these colloidal dispersions also makes them very attractive platforms for their application in several industries (cosmetics, pharmaceutical, or alimentary) on search of new eco-sustainable consumer products [43]. This research tries to present the first approach to the design of a ready-to-use insecticide formulation based on the solubilization of imidacloprid (0.003 wt%) in surfactantless emulsions obtained on the water/eugenol/ethanol ternary systems [44], which are expected to have some bioactivity against *Cimex lectularis* L. resistant to insecticides. This research is intended as a first step towards the design of new eco-sustainable ready-to-use pesticide formulations, taking advantage of the simplicity of the process leading to the surfactantless emulsions and their stability upon imidacloprid incorporation. However, its field application requires a careful examination of the possible hazards and risks for environmental and human health associated with these systems.

## 2. Results and Discussion

### 2.1. Physicochemical Characterization of Surfactantless Emulsions

**Figure 1:**
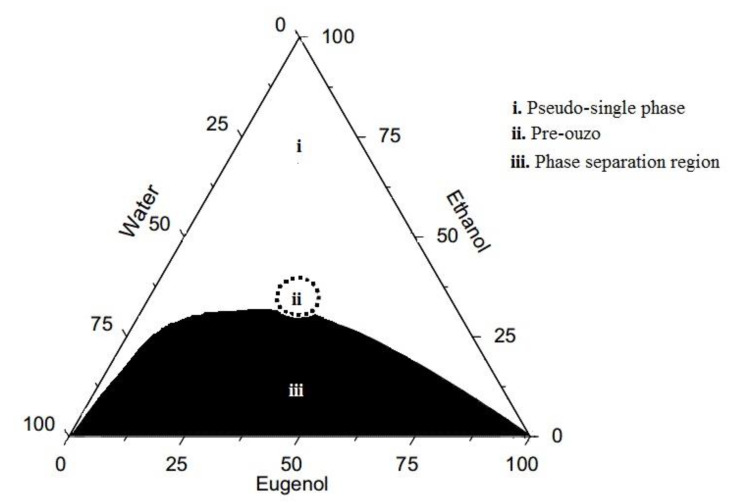
Phase diagram for the system water/eugenol/ethanol at 25 °C. The compositions of the different compounds are in weight fraction (wt%).

The phase diagram of the ternary water/eugenol/ethanol system was carefully studied in our previous research, and the existence of three different compositional regions was defined: (i) pseudo-single phase, (ii) pre-ouzo, and (iii) phase separation [44]. Figure 1 presents a simplified phase diagram of the ternary system, where the compositional regions corresponding to the three types of mixtures appearing for the ternary system are indicated.

Pseudo-single mixtures, appearing from eugenol-rich to water-rich regions, are macroscopically homogeneous (transparent) and thermodynamically stable samples. The pre-ouzo mixtures correspond to transparent pseudo-single mixtures, one enriched in eugenol, and the other rich in water. The phase separation region corresponds to cloudy multiphase mixtures [44]. This research is focused on the analysis of the ability of formulations, corresponding to the two macroscopically homogenous compositional regions (pseudo-singe phase and pre-ouzo), as a platform for solubilization and transport of a synthetic insecticide: imidacloprid. Dynamic light scattering (DLS) measurements were performed for the pseudo-single phase and pre-ouzo ternary mixtures of water, eugenol, and ethanol (notice that only transparent mixtures were analyzed by DLS) in the absence and presence of solubilized imidacloprid (final concentration of 0.003 wt%). For the sake of simplicity, the studied samples were divided into three different groups depending on each component proportion in the sample (see Table 1).

**Figure 2:**
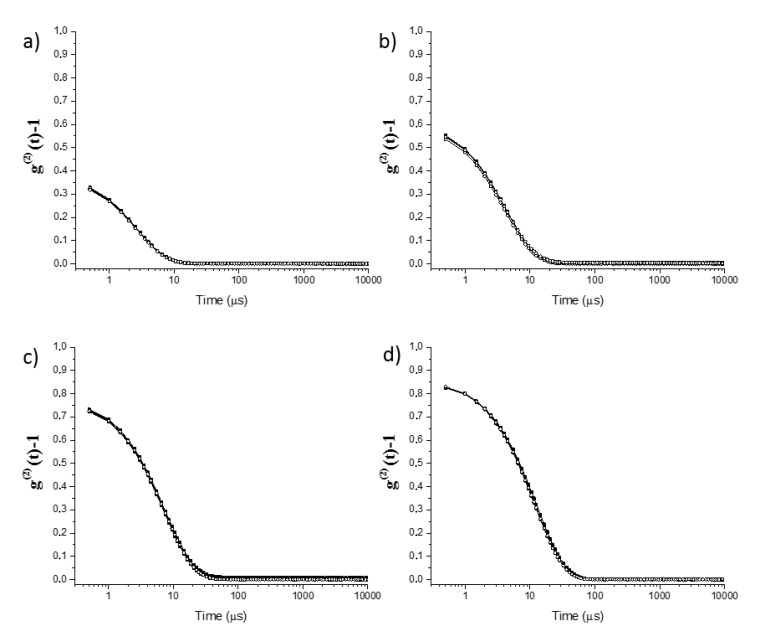
Dynamic light scattering (DLS) intensity autocorrelation functions for single-phase (**a**,**b**) and pre-ouzo (**c**,**d**) mixtures corresponding to path I (fixed water composition, 30 wt%) in absence (■) and presence (○) of imidacloprid (0.003 wt%). From a to d, there is a progressive substitution of ethanol for eugenol. Notice that the results correspond to the average of three independent measurements.

**Figure 3:**
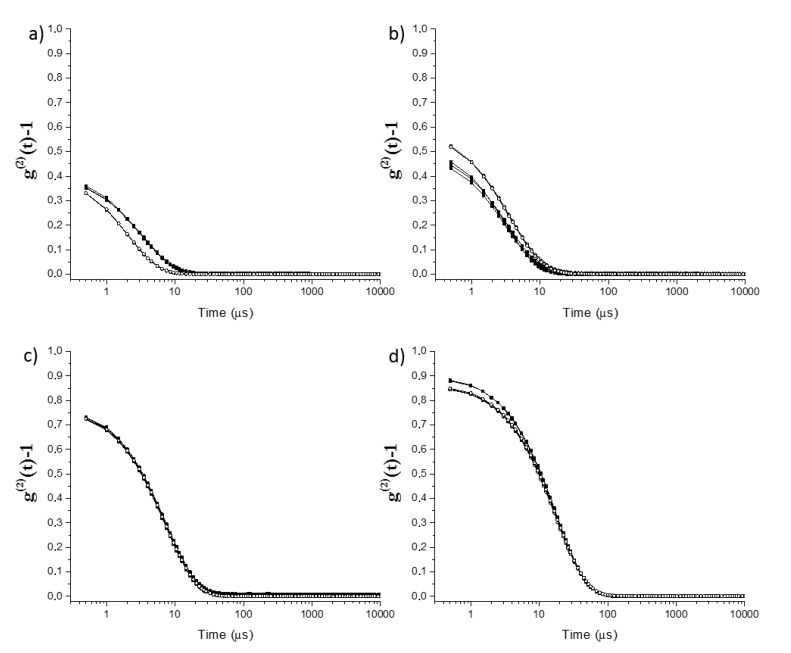
DLS intensity autocorrelation functions for single-phase (**a**,**b**) and pre-ouzo (**c**,**d**) mixtures corresponding to path II (fixed eugenol composition, 30 wt%) in absence (■) and presence (○) of imidacloprid (0.003 wt%). From a to d, there is a progressive substitution of ethanol for eugenol. Notice that the results correspond to the average of three independent measurements.

**Figure 4:**
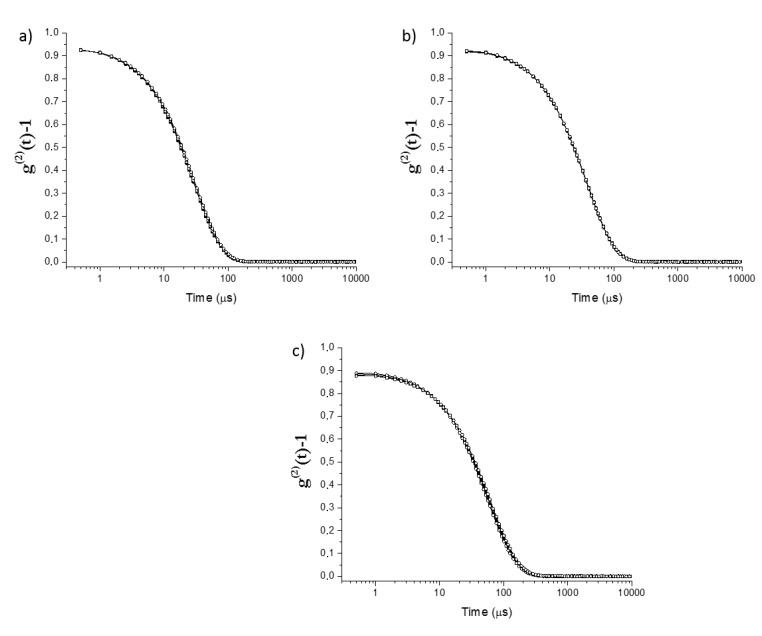
DLS intensity autocorrelation functions for pre-ouzo mixtures (**a**–**c**) corresponding to path III (fixed ethanol composition, 32.5 wt%) in the absence (■) and presence (○) of imidacloprid (0.003 wt%). From a to d, there is a progressive substitution of ethanol for eugenol. Notice that the results correspond to the average of three independent measurements.

Figure 2, Figure 3 and Figure 4 show the intensity autocorrelation functions obtained in DLS experiments for the ternary mixtures. The analysis of the intensity autocorrelation functions shows the worst correlation for the pseudo-single phase mixtures than for the pre-ouzo one, i.e., the intensity autocorrelation functions present lower values for pseudo-single phase mixtures than for pre-ouzo one. Moreover, the worst correlation in DLS experiments was found for mixtures in which the main component is ethanol, i.e., mixtures in which at least 50% of the total weight of the mixture is ethanol. This might have different origins: (i) low concentration of the scatters, and (ii) small size of the scatters. In both cases, it would be expected a poor contrast in DLS measurements. It is worth mentioning that the worst correlation in DLS experiments was found for mixtures in which the main component is ethanol, i.e., mixtures in which at least 50% of the total weight of the mixture is ethanol. Thus, considering the miscibility of ethanol either with eugenol or with water, this poor correlation (see Figure 2a, Figure 3a, Figure 2b and Figure 3b, respectively) suggests the possible formation of a pseudo-hydroalcoholic solution in which the droplets of the dispersed phase presents sizes comparable to the molecular scale, i.e., the dispersion within the continuous phase occurs almost at the molecular scale similarly to what happens in real solutions. Therefore, the determination of a real size for the dispersed droplets from the analysis of the intensity auto-correlation functions appears as physically unsound, making it necessary to discuss in terms of the mean relaxation time (note that the mean relaxation time is directly proportional to the apparent hydrodynamic diameter). As the pre-ouzo region is approached, and the compositions of the three compounds of the ternary mixture are closer, a better correlation was found as a result of the increase of the size of the dispersed droplets, which is associated with the approaching to the phase separation.

**Figure 5:**
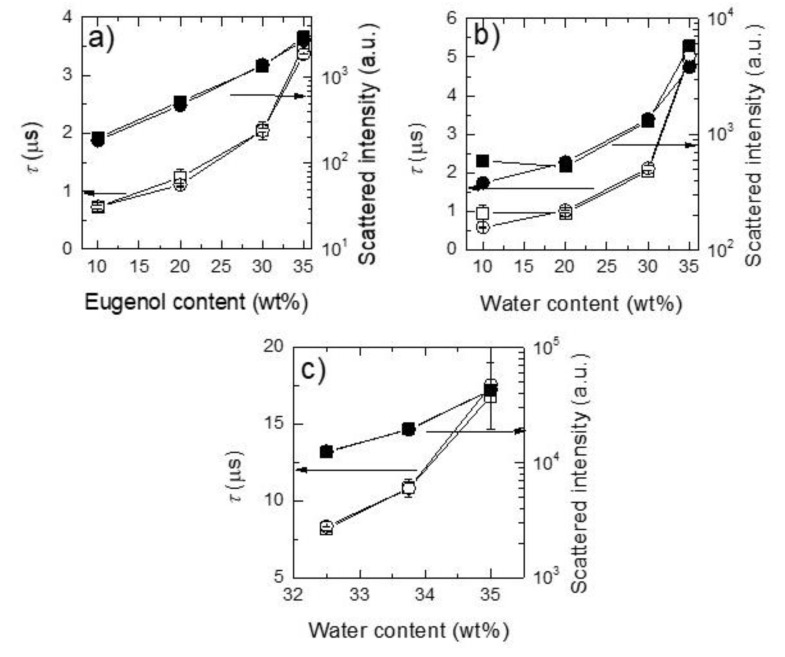
Mean relaxation times (left axis) and scattered intensities dependences on the composition of one of the mixture components for the different studied ternary mixtures. (**a**) For mixtures belonging to path I (water content constant), dependences on the eugenol content. (**b**) For mixtures belonging to path II (eugenol content constant), dependences on the water content. (**c**) For mixtures in path III (ethanol content constant), dependences on the water content. In all the panels: squares represent the bare ternary mixtures and circles represent the results for ternary mixtures upon imidacloprid incorporation (0.003 wt%). Open symbols correspond to data for mean relaxation time and solid symbols represent the data of scattered intensity. Lines are guides for the eyes.

The above discussion agrees with an increase of the mean relaxation time (see Figure 5), which is higher as the pre-ouzo region is closer owing to the increase in the size of the dispersed scatters. A similar conclusion may be drawn from the increase of the scattered intensity (see also Figure 5b) for the mixtures in the vicinity of the pre-ouzo region evidencing a better optical contrast between the dispersed and the continuous phases, which results from the increase of the droplets size and the worsening of the dispersion.

The inclusion of imidacloprid in the obtained dispersions (final concentration of 0.003 wt%) does not modify significantly the characteristics of the samples. Figure 2, Figure 3 and Figure 4 show for the sake of comparison the intensity autocorrelation function for mixtures without and with incorporated imidacloprid. 

The inclusion of the insecticide does not significantly change the intensity autocorrelation functions, with the mean relaxation times remaining almost the same after the imidacloprid inclusion (see Figure 5). The absence of any significant change may be assumed as a signature of the limited impact of the imidacloprid on the stability of the formulations. This is confirmed considering the values of the scattered intensity which do not show any significant modifications when imidacloprid is included. It is worth mentioning that even though the effect of imidacloprid in the mixtures is rather limited, the smallest effects were found for mixtures within the pre-ouzo region. This may prove that the formation of well-defined drops creates a favorable environment for the dispersion of a poorly soluble drug without influencing the stability of the formulations. 

### 2.2. Insecticide Activity of Formulations against a Resistant Strain of C. lectularius

This section analyzes the impact of surfactantless emulsions containing imidacloprid on a pyrethroid-resistant bed bug strain, and no comparison with any susceptible strain is included. This is because the exposure of a susceptible bed bug strain to a surfactantless emulsion containing a high dose of imidacloprid should not result in a significant change on mortality, or may increase a little such mortality if synergism is present in the formulation, compared to that found after the application of the same dose of imidacloprid following a different protocol. This is rationalized considering that the methodology of distribution of the insecticide does not modify their chemical nature, and consequently, their impact against susceptible insects should be considered similar independently of the protocol used for its application. Therefore, the discussion of this research is focused on the impact of the formulations on a resistant strain which presents many problems associated with its chemical control.

**Figure 6:**
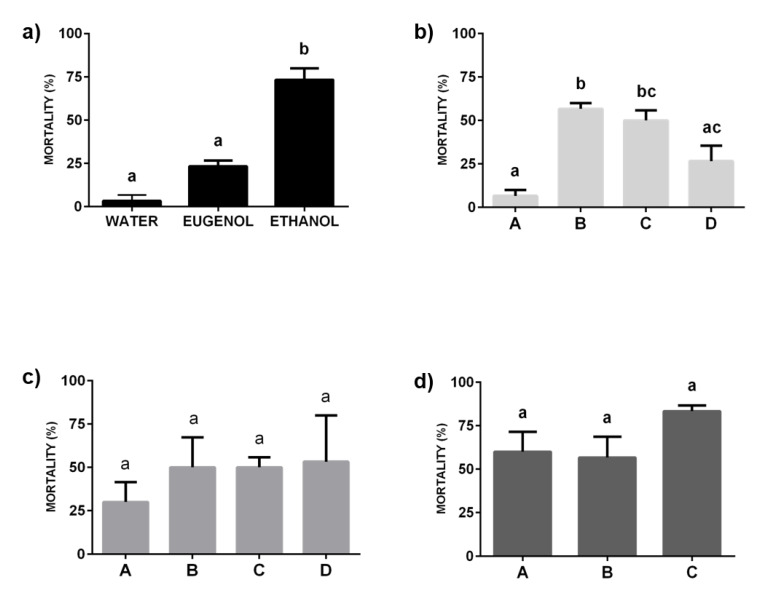
Mortality (Mean ± SEM) of adult resistant bed bugs (Retiro-R) against topical treatment with different formulations containing imidacloprid (0.003 wt%). Abbott’s formula was used to correct the mortality for control groups (data not shown). (**a**) Imidacloprid solubilized in the pure solvents: water, eugenol, and ethanol. (**b**) Imidacloprid included in mixtures corresponding to path I (samples containing water concentration constant). (**c**) Imidacloprid included in mixtures corresponding to path II (samples containing eugenol concentration constant). (**d**) Imidacloprid included in mixtures corresponding to path III (samples containing ethanol concentration constant). The lowercase letters above each bar evidence the significance in the difference between the results obtained for the different formulations, with the same letters indicating the absence of significant differences between treatments (*p* > 0.05).

The mortality produced by imidacloprid diluted in water, eugenol, ethanol, and their ternary mixtures was evaluated after topical and spray applications of the obtained formulations against adults of a *Cimex lectularis* L. pyrethroid-resistant colony. The results obtained upon application of solutions of imidacloprid in pure solvents against bed bugs evidenced that imidacloprid in ethanol presents a significantly higher insecticide efficacy than when the solvent is water or eugenol (F = 58.50; *p* = 0.0001), with the bed bug mortality being about the 73 ± 7% (Mean ± SEM) of the tested population (almost three-fold higher than the obtained for imidacloprid solutions in eugenol and almost 10-fold the mortality obtained for imidacloprid aqueous solutions as is shown in Figure 6a. 

**Figure 7:**
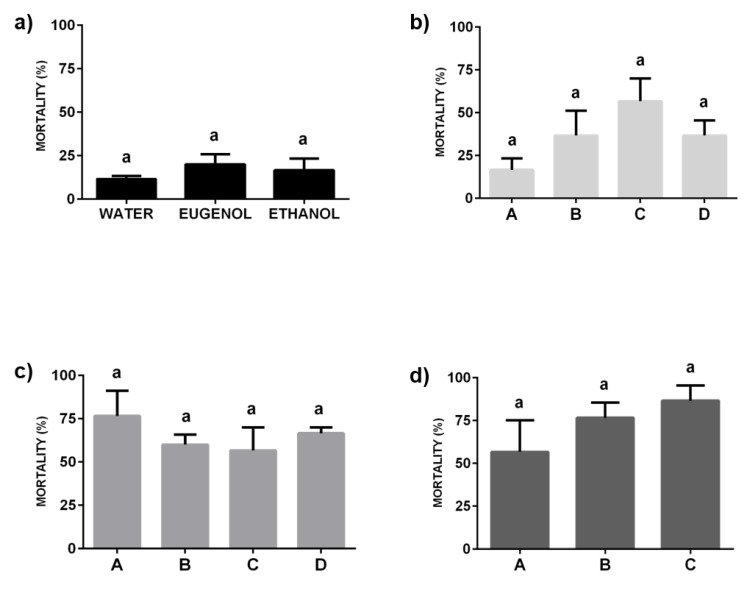
Mortality (Mean ± SEM) of adult resistant bed bugs (Retiro-R) against spray treatment with different formulations containing imidacloprid (0.003 wt%). Abbott’s formula was used to correct the mortality for control groups (data not shown). (**a**) Imidacloprid solubilized in the pure solvents: water, eugenol, and ethanol. (**b**) Imidacloprid included in mixtures corresponding to path I (samples containing water concentration constant). (**c**) Imidacloprid included in mixtures corresponding to path II (samples containing eugenol concentration constant). (**d**) Imidacloprid included in mixtures corresponding to path III (samples containing ethanol concentration constant). The lowercase letters above each bar evidence the significance in the difference between the results obtained for the different formulations, with the same letters indicating the absence of significant differences between treatments (*p* > 0.05).

The results obtained for the ternary mixtures after their topical application revealed significant differences in their efficacies, with an important dependence on the specific composition of the analyzed ternary mixture. The mixtures in the pre-ouzo region or close to such region evidence mortalities around 50% of the exposed insects (Figure 6). This is clearer from the analysis of the mortality induced by formulations with included imidacloprid belonging to paths II and III where no significant changes on the induced mortality were found with the change of the mixture composition (F = 0.39, *p* = 0.77 and F = 2.19, *p* = 0.19, for path II and path III, respectively). It is worth mentioning that the average mortality was found in all the cases to be higher for samples in path III, which present a similar weight fraction of the three components. This confirms the enhancement of the insecticide activity of the formulation belonging to the pre-ouzo region. The situation appears more complex for samples belonging to path I (water contain fixed, F = 15.67, *p* = 0.001), where significant differences in their insecticide efficacy were found, without any apparent explanation. In this path, the mortality produced by treatments B and C (>50%) was significantly higher than the mortality obtained in treatment A (6.67%). Despite the absence of any rational explication for the discussed differences, the scenario agrees with that found when the application using spray of the formulations is analyzed (Figure 7). For the latter bioassays, the mortality associated with treatments based in solutions of imidacloprid in pure solvents remains in all the cases in the range of 10–20% of the exposed insects and no significant differences were found between the mortality obtained as result of the application of insecticide solutions in different solvents (F = 0.65; *p* = 0.55).

The effect of the ternary mixtures containing imidacloprid upon application by spray is qualitatively similar to that found for mixtures applied topically, without any significant difference between the results obtained with samples corresponding to the same path (path I: F = 2.09, *p* = 0.18; path II: F = 0.71, *p* = 0.57; path III: F = 1.40; *p* = 0.32). Again, the samples belonging to paths II and III evidenced high levels of insecticide activity, with mortalities ranging between 50% and 80% of the insects exposed to the spray application (Figure 7c,d).

A better understanding of the relationships existing between the physicochemical properties of the formulations and their insecticide activity, the composition of samples, the scattered intensities, i.e., droplets size, and the mortality data may be obtained by analyzing in terms of a generalized linear model (GLM) for spray and topical treatments. It was found that the increase of the eugenol content in the samples is associated with an increase of the size of the aggregates (an increase of the scattered intensity) (*p* < 0.001), leading to formulations in which the imidacloprid may perform a better insecticide activity. Furthermore, the differences in mortality found in the treatments with formulations belonging to path I could be explained as a result of the low eugenol content. Table 2 summarized the data obtained from the analysis using the GLM.

The above results evidence that the use of formulation based in pre-ouzo mixtures containing imidacloprid (0.003%wt, Discriminant Dose (DD) ≈ 5 times × LD_99_ susceptible strain) against resistant bed bugs leads to significantly higher mortality than conventional formulations. This is clear considering that the resistance ratio to imidacloprid in the Retiro-R strain is 54-fold compared to the susceptible strain [18]. However, since the resistance to imidacloprid was even higher in several populations of *Cimex lectularius* sampled in the USA [45,46] and Argentina [18], the results obtained here should also be compared to those resulting from the evaluations with different bed bug strains belonging to a range of resistance ratios to give more consistency for their potential application in pest control and the management of resistance.

Furthermore, the topical application of imidacloprid solubilized in the ternary mixtures formulations provided the basis for reducing the applied dose down to a value 3.5 times lower than that the LD_50_, i.e., the dose that produces 50% of mortality in treated insects, corresponding to the resistant bed bugs [18]. Despite that insecticide effect produced with the surfactantless emulsions containing imidacloprid was not able to achieve 100% mortality, these results are promising for future evaluations in different experimental conditions. In a previous study, the effectiveness of many commercial products formulated with synthetic insecticides (pyrethroids and neonicotinoids dual products, carbamates) or green insecticides (diatomaceous earth, amorphous silica gel, EOs) was evaluated in a resistant and a susceptible bed bug strain [47]. The results showed that at the same labeled-dose, the mortality in resistant bed bugs was lower than 50% for any of these formulations, and also demonstrated the lack of options available to use for controlling bed bugs. Dang et al. [22] evaluated the efficacy of different insecticides using a range of solvents and surfaces in toxicity bioassays by direct contact against bed bugs. Their results showed an enhanced efficacy for oil-based imidacloprid treatments compared to the insecticide diluted in acetone. A similar effect is expected as a result of the presence of eugenol in the ternary mixtures, which enables the enhancement of the insecticide effect of imidacloprid when the formulations are directly applied on bed bugs. 

Eugenol is a monoterpene constituent of clove EOs [48], and its bioactivity as an insecticide and repellent has been demonstrated in several insect species [49,50,51,52]. Additionally, eugenol and other EOs could produce changes in the locomotor activity of triatomines (Heteroptera: Reduviidae) [53]. Reynoso et al. [36] observed that eugenol-hyperactivated nymphs of *Triatoma infestans* picked up more insecticide and then become intoxicated faster than non-hyperactivated nymphs when exposed to a permethrin-treated surface. Recently, Gaire et al. [54] evaluated the neurotoxic effect of 15 known EOs components on bed bugs. The topical application of eugenol did not give an exact mortality value, and the fumigant activity was intermediate compared to other monoterpenes such as carvacrol or thymol. However, a marked neuroinhibitory effect was observed in electrophysiological trials, even though the target sites for most EOs are still unknown, it has been proposed that eugenol acts on the octopamine receptors of the insect nervous system [55]. Singht et al. [56] demonstrated the reduced efficacy of commercial “eco-friendly” products based on mixtures of EOs compared to pyrethroid and neonicotinoid formulations in insecticide-resistant bed bugs. Their results showed that direct spraying on bed bug nymphs had an insecticidal effect of 90% after 10 days of treatment, and the mortality was 20–40% after 14 days of exposure to these EOs formulations. In the same study, the authors mentioned that direct spraying of a cedar oil emulsion (10% oil and 0.8% of 2, 6, 8-trimethyl-4-nonyloxypolyethylene oxyethanol as a surfactant) caused only 20% mortality in resistant insects and suggested that the concentrations of EOs in commercial formulations are too low to produce a significant insecticidal effect on bed bugs. Moreover, the toxic effect of eugenol at doses of 0.15 mL/cm^2^, 0.003 mL/43 cm^2^, and 10 µL/g has been observed in other insect species, such as *Periplaneta americana* [57], *Aedes aegypti* [58,59], and *Coptotermes formosanus Shiraki* [48], respectively.

It is worth mentioning that in the present research, the application of ternary mixtures containing imidacloprid leads to higher mortalities (acute toxicity) upon application by spray than when the application is performed topically. This result may be explained considering a better distribution of the samples within the insect groups when the application is performed using the spray. Furthermore, the results show clearly that the amount of eugenol is a critical parameter for the fabrication of effective platforms for the encapsulation of imidacloprid. This consideration is reinforced considering that the highest mortalities were found for mixtures belonging to pre-ouzo regions.

## 3. Materials and Methods

### 3.1. Chemicals

**Figure 8:**
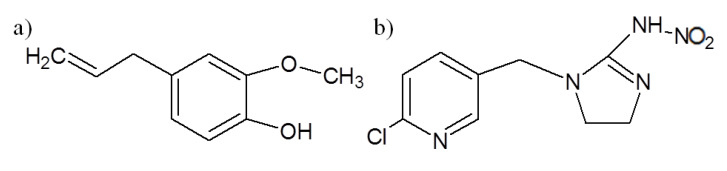
Molecular formulas of eugenol (**a**) and imidacloprid (**b**).

Eugenol (IUPAC Name 4-allyl-2-methoxyphenol) (99%-ReagentPlus^®^) and Imidacloprid (99%) (Figure 8) were purchased from Sigma-Aldrich (Saint Louis, MO, USA). Ethanol Absolute PRS (99.5%) was purchased from Panreac Química S.L.U (Barcelona, Spain). All the chemicals were used as received without further purification. The ultrapure deionized water (Milli-Q water) used was obtained by a multi-cartridge purification system AquaMAX^TM^-Ultra 370 Series (Young Lin Instrument Co., Ltd., Gyeonggi-do, South Korea), presenting a resistivity higher than 18 MΩ∙cm, and a total organic content lower than 6 ppm.

### 3.2. Samples Preparation

Samples were prepared in tubular glass vials (10 mL) following a procedure adapted from our previous publication [44]. Firstly, the binary mixtures of eugenol and ethanol with different concentrations were prepared. Then, the third component (water) was added dropwise until the ternary mixture with the final composition was obtained. The composition of the mixtures was calculated according to weighted amounts of the different components in weight percentage (%wt).

### 3.3. Design of the Experiments

Stable pseudo-single phase emulsions and pre-ouzo mixtures were used for the solubilization of imidacloprid (concentration of 0.003 wt%). The choice of the pseudo-single phase emulsions and pre-ouzo mixtures was done taking into consideration the phase diagram obtained in our previous research for the water/eugenol/ethanol ternary system [44].

Three different compositional paths were studied in this research, with this path corresponding to lines of the phase diagram in which the composition of one of the components remains fixed. The three paths studied are summarized in Table 1. The different studied mixtures were tested for imidacloprid solubilization which makes a careful examination of the behavior of the mixtures without the synthetic insecticide as reference necessary.

### 3.4. Dynamic Light Scattering (DLS)

Dynamic Light Scattering (DLS) experiments were performed using a Zetasizer Nano ZS Instrument (Malvern Instruments Ltd., Malvern, UK). The DLS measurements were performed at 25 ºC using the red line (wavelength, *λ* = 632 nm) of a HeNe laser in a quasi-backscattering configuration (scattering angle, *θ* = 173°). Before each measurement, the samples were filtered in a cleanroom using a 0.45 μm Nylon membrane (Millex^®^, Merck-Millipore, Burlington, MA, USA) to remove dust particles. The filtered samples were transferred to quartz measurement cells (Hellma 6030-OG Model). In DLS experiments, the normalized intensity autocorrelation function, g^(2)^(*q,t*), is obtained. This function presents an exponential-like decay when monodisperse scatters with Brownian motion are considered

(1) $$g^{\left(2\right)}\left(q,t\right)-1=\beta e^{-t/\tau }=\beta e^{-q^{2}D_{\mathrm{app}}t}$$

where *t* and *τ* are the time and the mean relaxation time of the intensity autocorrelation function, respectively, with the latter being related to the apparent diffusion coefficient of the scatters 1/*τ* = *D_app_q*^2^, where q=(4πnλ)sin(θ2) is the wavevector (with *n* being the continuous phase refractive index). In Equation (1), *β* is an optical coherence factor which generally assumes values close to 1, except for those cases in which the scattered intensity is low, which can be associated with the low size of the scatters, its low concentration, or the poor refractive index contrast between the scatters and the solvent. Assuming the scatters as spherical particles diffusing in a continuous Newtonian medium, it is possible to assume that the apparent diffusion coefficient is related to the apparent hydrodynamic diameter of the scatters $d_{H}^{\mathrm{app}}$ through the Stoke–Einstein relationship. However, considering the particular nature of the samples under study, the discussion will be done in terms of the mean relaxation time τ which can be assumed to be directly proportional to the size of the scatters [60].

### 3.5. Insects

Adult bed bugs from the insecticide-resistant strain Retiro-R were used for toxicity assessment. The Retiro-R strain was initiated with specimens collected in 2012 by pest control operators in a hotel of Buenos Aires (Argentina). The bed bug strain was conserved under laboratory rearing conditions without exposure to insecticides in the Centro de Investigaciones de Plagas e Insecticidas (UNIDEF-CONICET, Buenos Aires, Argentina) [18].

The rearing system consists of plastic jars covered with cotton voile on the top and corduroy fabric placed inside, which act as support, refuge, and oviposition site for bed bugs. Insects were fed with pigeon blood weekly according to a protocol approved by the Institutional Animal Care and Use Committee of CIPEIN (National System of Bioterium Nº: 1572/155). Culture conditions were 25 ± 1.5 °C, 40 ± 10% relative humidity (RH), 12:12 light: darkness (L:D) photoperiod [18]. Groups of 5th stage nymphs (*n* = 300) were separated from the rearing colony and fed with the same methodology explained above. After 7 days, newly molted adults (1:1, female and male) were obtained and used for toxicity bioassays 5 days later.

### 3.6. Toxicity Evaluation

Topical and spray bioassays in adult bed bugs were performed to test the toxicity of imidacloprid solubilized in the three solvents (water, eugenol, or ethanol) and the studied ternary mixtures (pseudo-single phase and pre-ouzo mixture). The first step for these studies was the determination of the susceptibility baseline of imidacloprid in adult bed bugs, evaluating the effect of imidacloprid solutions in the three solvents (water, eugenol, or ethanol). Then, the different ternary mixtures containing imidacloprid were tested against insecticide-resistant bed bugs. Control treatments were performed with single solvents and ternary mixtures without imidacloprid.

#### 3.6.1. Topical Application Bioassay

The topical application of imidacloprid dissolved in water, eugenol, and ethanol was made to determine the susceptibility baseline and to standardize the technique and applied dose [18]. For this purpose, 0.2 µL of imidacloprid solution was dropped onto the dorsal abdominal surface of insects using a micro-applicator PB600-1 (Hamilton Co., Reno, NV, USA). In order to discriminate the effect of the solvent and that of the insecticide, the same dose of the solvents was applied to the control. Our previous studies showed values of LD_50_ and LD_99_ (CL 95%) for imidacloprid solutions in ethanol in adult bed bugs of HH-S (the reference susceptible strain) of 0.04 and 1.37 ng per insect (Mean ± CL 95%), respectively. This allows establishing the susceptibility baseline with the criteria of Discriminant Dose (DD ≈ 5 times × LD_99_ = 0.003%wt) [61].

Therefore, a single dose (DD = 0.003%wt) was applied with the methodology mentioned above. Immediately after the topical application, insects (in groups of 10) were placed in a clean plastic vial and kept under the same rearing conditions as the native colonies. At least, three independent replicates were made for each tested sample (*n* = 100). Mortality data was recorded after 24 h by placing the treated insects in the center of a filter paper (110 mm) and observing the bed bugs that died or exhibited intoxication symptoms (locomotor incoordination or standing without movement) during 1 min. Finally, to evaluate the mortality of the ternary mixtures against *Cimex lectularius* L., the same methodology described above was used, and at least three independent replicates were made for each studied mixture (*n* = 300).

#### 3.6.2. Spray Application Bioassay

The toxicity effect of ternary mixtures containing imidacloprid was also tested upon spray application. For these, the susceptibility baseline was fixed after spray application of imidacloprid solutions in water, eugenol, or ethanol at a unique dose (0.10 µg/cm^2^), equivalent to the Discriminant Dose obtained from the topical bioassay. The same dose was applied for the evaluated ternary mixtures. For this type of experiment, groups of 10 adults (1:1, male:female) were placed inside of the glass ring (10 cm^2^) with the bottom side covered with cotton voile and attached with a rubber band. This was evaluated as the most suitable condition to keep 10 bed bugs adequately dispersed, avoiding their aggregation. Using a clear plastic bottle (20 mL) with a pump dispenser containing 2 mL of each ternary mixture was once-shot discharged on the bed bug groups from a 20 cm distance (notice that the optimal distance for spray application was determined after screening tests consisting in spraying different solvents and emulsions from three different distances (5, 10, 20 cm) from the open pump dispenser that allows covering a surface of 10 cm^2^. The distribution of different formulations discharged on a piece of fabric with the dimensions mentioned above was observed under a stereoscopic microscope to ensure the equal distribution within this surface). Immediately after the application, insects in groups were placed in a clean plastic vial and kept under the same rearing conditions as the native colonies. At least three independent replicates were made for each ternary mixture (*n* = 280). Mortality data were obtained following the same protocol used for the topical bioassays.

### 3.7. Statistical Analysis

Mortality data were corrected using Abbott′s formula when the mortality ranged between 10% and 20% of insects tested in control groups [62]. Mortality data were analyzed by one-way ANOVA, followed by Tukey test to compare the differences between the mean mortality values (ternary mixtures in each path or imidacloprid diluted in each solvent). A value of *p* < 0.05 was considered statistically different.

The correlation between the physicochemical characteristics of the evaluated formulations and the mortalities found in topical and spray bioassays were evaluated based on a generalized linear model (GLM). The GLMs followed Poisson distribution errors, and log was the link function with water, eugenol, ethanol, size, and scattered intensity as factors. Model selection was based on the Akaike information criteria and we include variables that presented significant regressions alone. If two variables were correlated >0.80, those variables that explained less were excluded from further modeling. The standardized residuals were plotted against fitted values (to verify lack of fit of the residuals) and normality checked with quantile–quantile plots (data quantiles against normal quantiles). Since the Poisson models showed over-dispersed data, we assumed a negative binomial distribution of the data for the analysis [63]. The differences were considered significant at a *p*-value of <0.05. All statistical analyses were performed with the software R-Studio (version 1.1.383, R-Studio, Boston, MA, USA) [64] and conducted using the lme4 [65], MASS, and multcomp packages (version 1.4-13, Cran-R-project, Zurich, Switzerland) [66].

## 4. Conclusions

Ternary systems composed of water, eugenol, and ethanol can result in pseudo-single phase (pseudo-hydroalcoholic solutions), pre-ouzo, and phase separation mixtures depending on the compositional range, with the former two being useful for the solubilization of a poorly water-soluble synthetic insecticide (imidacloprid). The incorporation of such insecticide does not impact detrimentally on the stability of the colloidal dispersions which present a potential interest in the development of formulations with insecticide activity. It is true that even though both hydroalcoholic solutions and pre-ouzo dispersions can be used as solubilizing platforms for imidacloprid, the existence of a localized structure within the droplets of the pre-ouzo dispersions makes them a more suitable environment for the dispersion of the insecticide, improving its availability during the formulation application.

This is particularly important because the preliminary test of these formulations against an insecticide-resistant *Cimex lectularius* colony have evidenced mortalities of around 80–85% of the individuals, which is generally enhanced as the increased amount of eugenol in the formulation. This suggests a possible synergism between the essential oil and the synthetic insecticide on the bioactivity of the formulations. Therefore, even though the extension of the present research is limited to a very specific formulation with a fixed insecticide concentration, it can be assumed that pre-ouzo mixtures of the ternary water/eugenol/ethanol system containing imidacloprid present a potential interest for developing formulations for effective topical and spray treatments against resistant bed bugs which may enable overcoming the resistance found when conventional formulations containing pyrethroids are used.

However, the real field application of these formulations requires a careful examination of several additional aspects. Firstly, this study has only analyzed the performance of formulations containing a fixed imidacloprid concentration, which provides information useful on the possible development of this type of formulation. However, a closer examination to the dose–response of the target insect against formulations containing different imidacloprid concentrations to optimize the composition or formulations to maximize the efficiency is required. Furthermore, the study of the residual effect associated with the use of the formulations is also critical. This is particularly important because bed bugs have a gregarious mode of life, living hidden in shelters of walls and mattresses, having the ability to survive through prolonged starvation [67]. These are factors that should be taken into account when designing control strategies and appropriate use of insecticides.

## Figures and Tables

**Table 1:** Composition of the different ternary mixtures studied in this research.

Paths ^1^	Sample	Region	Water (wt%)	Ethanol (wt%)	Eugenol (wt%)
I	a	Pseudo-single	30	60	10
I	b	Pseudo-single	30	50	20
I	c	Pre-Ouzo	30	40	30
I	d	Pre-Ouzo	30	35	35
II	a	Pseudo-single	10	60	30
II	b	Pseudo-single	20	50	30
II	c	Pre-Ouzo	30	40	30
II	d	Pre-Ouzo	35	35	30
III	a	Pre-Ouzo	32.5	32.5	35
III	b	Pre-Ouzo	33.75	32.5	33.75
III	c	Pre-Ouzo	35	32.5	32.5

^1^ All the samples are ternary mixtures with the compositions of the different compounds presented in weight fraction (wt%). The region indicates the compositional region of the considered mixture: pre-ouzo and pseudo-single phase. Path I (constant water concentration), path II (constant eugenol concentration), and path III (constant ethanol concentration).

**Table 2:** Results obtained from the analysis of the experimental results in terms of generalized linear models (GLM) for spray and topic treatments.

Variable	Factors	Parameter	D.F ^1^.	Z Value
Spray mortality	Intercept	2.61	1	7.63 **
	Eugenol	0.04	1	3.86 **
	SI ^2^	6.75 × 10^−6^	1	1.06
Topic mortality	Intercept	2.54	1	4.79 **
	Eugenol	0.04	1	2.19 *
	SI ^2^	1.15 × 10^−5^	1	1.12

^1^ D.F.: degrees of freedom. ^2^ SI: scattered intensity. Significance codes: * < 0.05. ** < 0.001.
